# Intralipid Therapy for Inadvertent Peripheral Nervous System Blockade Resulting from Local Anesthetic Overdose

**DOI:** 10.1155/2015/486543

**Published:** 2015-02-12

**Authors:** Ihab Kamel, Gaurav Trehan, Rodger Barnette

**Affiliations:** Temple University School of Medicine, 3401 N. Broad Street, 3rd Floor Outpatient Building, Zone B, Philadelphia, PA 19140, USA

## Abstract

Although local anesthetics have an acceptable safety profile, significant morbidity and mortality have been associated with their use. Inadvertent intravascular injection of local anesthetics and/or the use of excessive doses have been the most frequent causes of local anesthetic systemic toxicity (LAST). Furthermore, excessive doses of local anesthetics injected locally into the tissues may lead to inadvertent peripheral nerve infiltration and blockade. Successful treatment of LAST with intralipid has been reported. We describe a case of local anesthetic overdose that resulted in LAST and in unintentional blockade of peripheral nerves of the lower extremity; both effects completely resolved with administration of intralipid.

## 1. Introduction

Local anesthetics are well established in the practice of anesthesia. Although local anesthetics have an acceptable safety profile, significant morbidity and mortality have been associated with their use. Inadvertent intravascular injection of local anesthetics and/or the use of excessive doses have been the most frequent causes of local anesthetic systemic toxicity (LAST). Excessive doses of local anesthetics injected at the surgical site may lead to inadvertent peripheral nerve infiltration and blockade. We describe a case of local anesthetic overdose that resulted in early manifestations of LAST and unintentional sensory and motor blockade of peripheral nerves of the lower extremity. The concurrent sensory and motor blockade of the lower extremity completely resolved with intralipid that was administered to treat early symptoms of LAST.

## 2. Case Report

A 51-year-old, 163 cm, 74 kg, ASA-2 female was scheduled for posterior colpoperineorrhaphy and transobturator sling insertion. Her past medical history was significant for asthma, urinary incontinence, and multiple uneventful cesarean deliveries. The patient received general endotracheal anesthesia using propofol, vecuronium, sevoflurane, and fentanyl. Surgery was performed with the patient in the lithotomy position. Shortly prior to the conclusion of the procedure 80 mL of 0.5% bupivacaine with epinephrine was injected by the surgeon into the surgical incision site for postoperative analgesia. The surgical procedure lasted for 90 minutes.

In the postanesthesia care unit (PACU) the patient experienced dizziness, agitation, posturing, and oculogyric symptoms and stated that she “felt funny.” The patient further stated that she could not feel or move her left lower extremity and that it felt “numb.” On examination the left lower extremity revealed nonflaccid loss of motor power (1/5) in the hip flexors and adductors and inability to articulate the left knee joint. She had decreased touch sensation over the anterior and medial aspect of the left thigh and the left leg (L2–L5 dermatomes). The neurology service was immediately consulted to evaluate the patient for the possibility of stroke. Approximately 20 minutes after arriving at PACU, the patient was treated with 100 mL of intralipid 20% I.V. fat emulsion (Baxter Healthcare Corporation, Deerfield, IL) over one minute, followed by 400 mL of intralipid 20% over 20 minutes. Immediately upon completion of the initial intralipid loading dose, the patient's overall condition improved; the intermittent oculogyric symptoms resolved and she became less agitated and stated that she “felt better.” During the subsequent intralipid infusion the patient stated that she could now feel her left lower extremity and slight knee movement was noted. On examination, the patient regained sensation over the medial and anterior left thigh and the left leg. Motor evaluation revealed return of motor power to left hip flexors and adductors and the ability to articulate the left knee joint. Following completion of the infusion the patient had restoration of motor function and sensation to the left lower extremity. The neurology service arrived approximately 30 minutes after the patient's arrival at PACU and concluded that her left lower extremity symptoms were likely due to the excessive dose of local anesthetic injected during the procedure, rather than a cerebrovascular accident. They also noted that the patient's left lower extremity weakness and sensory loss were significantly improving with intralipid injection. The patient maintained awareness and spontaneous ventilation with a SpO_2_ of 98% or higher throughout the event. EKG showed sinus rhythm without evidence of ectopy or arrhythmia. A 12-lead EKG revealed no change in comparison to the preoperative EKG. Prior to administration of intralipid, she experienced one episode of hypotension that was successfully treated with 100 mcg of phenylephrine. The patient continued to complain of left lower extremity parasthesias; however these had resolved completely by the next morning. The patient was discharged home on the first postoperative day without sequelae.

## 3. Discussion

Successful intralipid treatment of cardiovascular and central nervous system (CNS) local anesthetic toxicity has been reported [1–5]. This patient received approximately twice the maximum recommended dose of bupivacaine and appeared to experience early manifestations of central nervous system local anesthetic toxicity. The patient inadvertently received 80 mL of 0.5% bupivacaine (400 mg) instead of 0.25% (200 mg). This occurred because the operating room technician did not dilute the 0.5% bupivacaine solution as instructed. The onset of symptoms occurred gradually as the bupivacaine injected at the surgical site was absorbed into the blood stream. Because of the gradual and partial absorption of bupivacaine and epinephrine into the blood stream the patient did not experience acute full-blown manifestations of LAST and tachycardia. These findings prompted rapid treatment and all signs and symptoms of LAST resolved with administration of intralipid. The use of intralipid upon identifying early premonitory symptoms and signs can prevent further clinical manifestations of CNS toxicity and progression to cardiovascular toxicity [2, 6–8]. Atypical presentation has been reported in approximately 40% of patients experiencing LAST [7], and cases in which patients had minimal premonitory signs of CNS toxicity, such as oculogyric movement and agitation, have progressed to cardiac arrest [9]. Airway management and circulatory support have been reported to be crucial to successful treatment of LAST. However, in this case, the patient maintained adequate respiratory and cardiovascular functions.

In this patient, local anesthetic overdose was associated with a new onset unilateral lower extremity peripheral nerve block. While gradual systemic absorption of the excessive bupivacaine dose is responsible for LAST, diffusion of the excessive bupivacaine and local infiltration of surrounding tissues could explain the concomitant peripheral nerve blockade of the left femoral and obturator nerves. Postoperative unilateral lower extremity peripheral nerve blockade due to local anesthesia injection during transobturator sling placement has been reported [10].

The rapid temporally associated resolution of both sensory and motor symptoms in the left lower extremity during the administration of intralipid in the absence of any other interventions raises the possibility of a cause-effect relationship. This observation deserves further investigation in regard to the effect of intralipid on peripheral nerve local anesthetic blockade. Despite the plethora of reports and studies on intralipid use to treat LAST, our review of the current literature did not reveal any reports on the effect of intralipid on peripheral nerve local anesthetic blockade. The ability to reverse peripheral nerve blockade could allow early neurological examination and potentially prevent delays in diagnosis, thus reducing the risk of permanent neurological damage.

The mechanism of action of intralipid is incompletely understood. It is believed that intralipid provides an intravascular compartment for lipid soluble drugs [11]. Intralipid may affect metabolism, distribution, and displacement of local anesthetics from receptors into lipids within the tissues [4, 11] and counteract the inhibitory effect of bupivacaine on fatty acid transport at the inner mitochondrial membrane [11]. Although the safety profile of acute lipid emulsion infusion is not completely understood, there are no published reports of adverse outcomes [5].

## 4. Conclusion

We report a case in which administration of intralipid to treat LAST resulted in complete and rapid resolution of concomitant peripheral nerve blockade symptoms caused by local infiltration of bupivacaine overdose. Intralipid could be used to reverse prolonged or inadvertent peripheral nerve blocks allowing for earlier neurological evaluation.
